# Acceptance and perceived efficacy of intraoral versus extraoral photobiomodulation for oral mucositis prevention in pediatric patients

**DOI:** 10.1007/s00520-026-11227-3

**Published:** 2026-09-23

**Authors:** Isabela de Oliveira Plugge Freitas, Giovanna Leal Klein Renz, Isabela Schuartz, Mara Albonei Dudeque Pianovski, Laurindo Moacir Sassi, Bruna da Fonseca Wastner, Melissa Rodrigues de Araujo

**Affiliations:** 1 Federal University of Paraná, Curitiba, Brazil; 2 Erasto Gaertner Hospital, Curitiba, Brazil; 3 National Institute of Science and Technology in Translational Biophotonics in Dentistry (INCT-BIOFOTO BUCAL), Porto Alegre, Brazil

**Keywords:** Low-level light therapy, Oral mucositis, Pediatric assistants, Medical oncology

## Abstract

**Objective:**

To compare acceptance, tolerability, and perceived efficacy of extraoral (EO) and intraoral (IO) photobiomodulation therapy (PBMT) for oral mucositis (OM) prevention in pediatric oncology patients.

**Methods:**

Caregivers of 32 pediatric patients undergoing chemotherapy completed a structured questionnaire after their children experienced both intraoral and extraoral PBMT for oral mucositis prevention during different chemotherapy cycles within a randomized clinical study. Device preference, discomfort, perceived pain control, and reasons for preference were descriptively analyzed.

**Results:**

EO-PBMT was preferred by 65.6% of respondents, whereas 34.4% preferred IO-PBMT. Preference for EO-PBMT was mainly associated with greater comfort and avoidance of mouth opening. Discomfort was reported by 43.8% during IO-PBM and by 6.3% during EO-PBMT, with a statistically significant difference between the modalities (*p* = 0.001). IO-PBMT was more often perceived to provide better pain control (34.4%), whereas 12.5% favored EO-PBMT.

**Conclusion:**

EO-PBMT demonstrated greater caregiver acceptance and lower treatment-related discomfort than IO-PBMT. Although caregivers more frequently perceived IO-PBMT as providing better pain control, these subjective perceptions did not correspond to differences in the clinical occurrence or severity of OM between treatment modalities. EO-PBMT therefore represents a feasible and well-accepted approach for supportive care in pediatric oncology.

**Supplementary Information:**

The online version contains supplementary material available at https://doi.org/10.1007/s00520-026-11227-3.

## Introduction

Oral mucositis (OM) is a frequent, painful complication of cancer therapy in pediatric patients [1, 2], affecting 50–80% of children depending on oncologic treatment [2–5]. It presents as erythema, erosion, or ulceration, with or without pseudomembrane, developing 3–10 days after chemotherapy and persisting up to 3 weeks [6]. Methotrexate (MTX), used in acute lymphoblastic leukemia, non-Hodgkin lymphoma, and osteosarcoma, is strongly associated with high OM incidence [7].

OM severity ranges from mild to severe; pediatric patients are more susceptible due to higher mitotic activity and epithelial turnover [3, 4, 8]. Severe cases involve ulceration and secondary infections that may progress to sepsis [3]; oral and gastrointestinal involvement can also impair swallowing and nutrition, affecting quality of life and treatment continuity [6].

Photobiomodulation therapy (PBMT) has been recommended by the Multinational Association of Supportive Care in Cancer (MASCC) and International Society of Oral Oncology (ISOO) for OM prevention and management in adult cancer patients [9–12]. However, evidence in pediatric oncology remains limited, with no established optimal dosimetric parameters or standardized protocols [13].

Alternative delivery approaches have therefore been explored in pediatric settings. Extraoral (EO) PBMT is non-invasive, requiring no mouth opening or mucosal contact, reducing discomfort and infection risk [14–16], while enabling irradiation of oropharyngeal regions and, with multi-beam devices, shorter application times [16, 17]. Intraoral (IO) PBMT, in contrast, requires multiple application points and greater cooperation, making it more technique-sensitive and less feasible in pediatric practice [18].

Assessment of treatment-related discomfort and perceived benefit is essential in OM management, though self-reported measures are often limited in pediatric populations, making caregiver-reported outcomes a relevant complementary source of information [2].

Despite the increasing use of PBMT for OM management, evidence in pediatric oncology remains limited, particularly regarding the comparative feasibility and acceptability of different delivery approaches. Moreover, caregiver-reported outcomes, which are especially relevant in pediatric settings, remain underexplored in this context. Therefore, this study aimed to compare caregivers’ perceptions of intraoral and extraoral PBMT by evaluating caregiver-reported treatment acceptance, treatment-related discomfort, preference, and perceived efficacy following exposure to both treatment modalities. The present manuscript specifically focuses on caregiver-reported outcomes.

## Materials and methods

### Study design and ethical approval

This study was designed as a prospective, randomized, unblinded, crossover clinical study approved by the Institutional Human Research Ethics Committee (Protocol No. 4.866.526). Written informed consent was obtained from all participants’ legal guardians. During successive chemotherapy cycles, participants received both intraoral and extraoral PBMT according to a randomized treatment sequence. At the end of study participation, caregivers completed a structured questionnaire evaluating treatment acceptance. The present manuscript was prepared in accordance with the CONSORT 2025 statement for randomized trials, and reports these caregiver-reported outcomes, which were collected within the context of the clinical study.

### Participants

A convenience sample of pediatric patients (≤ 18 years) of both sexes diagnosed with malignant neoplasms (leukemia, lymphoma, or osteosarcoma) and undergoing high-dose methotrexate (HDMTX), either alone or in combination with the MADIT protocol (methotrexate, ARA-C, and dexamethasone), was included.

#### Randomization and study procedures

Participants were randomly assigned through simple electronic randomization (random.org) to receive either intraoral (IO) or extraoral (EO) PBMT before chemotherapy for the prevention of OM. Random sequence generation and treatment allocation were performed by a researcher not involved in PBMT administration, ensuring that clinicians delivering the intervention were not responsible for assignment. Each chemotherapy cycle included only one PBMT modality (IO or EO). In the subsequent cycle, with cycles typically spaced 3 weeks apart according to the institutional chemotherapy protocol, patients received the alternate PBMT modality, ensuring that all participants were exposed to both interventions, allowing within-subject (paired) comparisons.

PBMT was performed prior to HDMTX infusion and applied daily until hospital discharge. The laser devices used in this study were periodically calibrated by the manufacture.

#### Outcomes

The primary outcomes were caregiver-reported treatment acceptance, perceived efficacy, and discomfort, assessed through a structured questionnaire after completion of both PBMT modalities.

Secondary outcomes included the clinical occurrence and severity of oral mucositis (OM), assessed by a calibrated examiner using the World Health Organization (WHO) grading scale (Grade 0–IV) [19], and pain intensity measured using an Eleven-Point Numeric Rating Scale (NRS-11, 0–10) [20], for children who were able to self-report.

Throughout this manuscript, the term “perceived efficacy” refers exclusively to caregivers’ subjective impressions reported via questionnaire, and is conceptually distinct from clinical efficacy, which refers to objectively measured outcomes such as OM occurrence, severity, or grading, assessed separately as secondary outcomes.

### Questionnaire assessment

After completing both PBMT modalities, parents or caregivers completed a self-administered nine-item online questionnaire evaluating device preference, discomfort, perceived pain control, and reasons for preference.

### Interventions

#### Intraoral PBMT (IO-PBMT)

In the IO-PBMT group, the device and dosimetric protocol are described in Table 1. PBMT was applied at 42 intraoral points distributed six points on the right and left buccal mucosa, three points on the right and left lateral borders of the tongue, three points on the ventral surface of the tongue (right and left), two points on the right and left floor of the mouth, four points on the upper lip, four points on the lower lip, four points on the upper and lower labial mucosa, and two points on the right and left soft palate. This protocol was adapted from MASCC/ISOO guidelines [21]. Intraoral irradiation required approximately 7 min of light delivery, with a total clinical time ranging from 20 to 40 min.

**Table 1:** Comparison of intraoral and extraoral photobiomodulation devices and application protocols used for OM prevention

Parameters	Intraoral PBMT	Extraoral PBMT
Device	Diode laser—AlGaInP (Therapy EC, DMC, Brazil)	Diode laser—AlGaInP (E-Light IRL, DMC, Brazil)
Wavelength (nm)	660	660
Power (mW)	100	100
Spot size (cm^2^)	0.098	Central emitters: 0.029; lateral emitters: 0.233
Fluence (J/cm^2^)	10.16	Lateral emitters: 4.30; central emitters: 34.50
Irradiance (W/cm^2^)	1.02	Central: 3.45; lateral: 0.43
Energy/emitter (J)	1	1
Number of emitters	1	8
Application sites	Buccal mucosa, tongue (lateral and ventral), floor of mouth, lips, labial mucosa, soft palate	Orbicularis oris, left buccinator, right buccinator muscle
Total of Points	42	24
Distance from tissue (mm)	Contact	Contact

#### Extraoral PBMT (EO-PBMT)

In the EO-PBMT group, the device and dosimetric protocol are described in Table 1. The device includes four lateral emitters delivering 4.30 J/cm^2^ and four central emitters delivering 34.50 J/cm^2^. PBMT was applied extraorally to three anatomical regions: the orbicularis oris muscle and the left and right buccinator muscles. Each region received 10 s of irradiation, totaling 30 s per session. As the device operates with eight emitters simultaneously, 24 points were irradiated per session. Total appointment time ranged from 5 to 10 min.

### Procedures common to both groups

For both study groups, PBMT was initiated before HDMTX infusion as a prophylactic intervention before chemotherapy and was repeated once daily throughout hospitalization, following protocols from the literature [24, 35]. Treatment was continued until hospital discharge because patients receiving HDMTX remain hospitalized for hyperhydration, urine alkalinization, leucovorin rescue, laboratory monitoring, and confirmation of methotrexate clearance [1, 39, 40]. The number of PBMT sessions corresponded to the length of hospitalization, with a median (range) of 4 (3–6) sessions per cycle, which was comparable between the modalities.

### Management of oral mucositis lesions

Patients who developed OM during the evaluation period received therapeutic PBMT applied directly to the lesions using the same intraoral device. The following parameters were used: wavelength of 660 nm, average power of 100 mW, energy of 0.5 J per point, and energy density of 5.08 J/cm^2^.

### Survey assessment

After completing two chemotherapy cycles, one with intraoral PBMT (IO-PBMT) and another with extraoral PBMT (EO-PBMT), in any order, participants were invited to complete the survey. Data collection procedures were adapted according to the child’s developmental level. Pediatric patients capable of self-reporting completed the questionnaire independently. For those unable to do so, parents or legal guardians assisted by reading the questions aloud and recording responses based on their observations of the child’s reactions during PBMT sessions.

The self-administered questionnaire consisted of nine items designed to assess perceptions of PBMT modalities. The instrument evaluated: preferred PBMT modality (IO vs EO) and reasons for preference; perceived treatment effectiveness; occurrence of discomfort during application; perceived differences in pain control; and perceived impact on quality of life. Additional items explored caregiver-reported impressions when direct patient responses were not feasible (supplementary material).

This manuscript reports an exploratory analysis of caregiver-reported treatment acceptance and perceived efficacy, derived from participants enrolled in a larger randomized clinical trial comparing the clinical efficacy of intraoral and extraoral PBMT. As the present study focuses exclusively on caregiver-reported perception rather than clinical efficacy, no a priori sample-size calculation was performed for this questionnaire-based component.

### Statistical analysis

Descriptive statistics were used to summarize the data. Continuous variables were expressed as means and standard deviations or medians, as appropriate, while categorical variables were presented as frequencies and percentages. Age was categorized into three groups: < 5 years, 5–10 years, and > 10 years.

Although the intervention followed a crossover design, the post-treatment questionnaire was administered as a single cross-sectional evaluation. McNemar’s test was used only for the paired binary outcome (presence of discomfort between intraoral and extraoral laser applications within the same patient). For other survey variables, Fisher’s exact test was applied as an exploratory analysis to assess associations between laser preference and categorical variables (pain control, mucositis, discomfort, and reasons for preference). Fisher’s test was chosen due to the small sample size and contingency table cells with expected frequencies < 5. All analyses were performed using Jamovi (version 2.6.44), with p < 0.05.

## Results

The questionnaire was distributed to 36 parents or caregivers of pediatric oncology patients who had received both intraoral and extraoral PBMT during different chemotherapy cycles between September 2022 and June 2025. A total of 32 responses were included in the final analysis.

Regarding age distribution, 18.8% (*n* = 6) of participants were aged ≤ 5 years, 34.4% (*n* = 11) were between 5 and 10 years, and 31.3% (*n* = 10) were older than 10 years, while age information was missing for 15.6% (*n* = 5). Oral mucositis occurred in 50.0% of patients, while 34.4% did not develop the condition; 15.6% are missing data. Missing age and oral mucositis data corresponded to the same respondents and resulted from an inability to link certain online questionnaire responses to participants’ clinical records, rather than from a modality, or cycle, related pattern of missingness. OM was observed across all age groups (Table 2).

**Table 2:** Patient’s age and the occurrence of oral mucositis

Age	OM occurrence	*N*	%
≤ 5 years	Yes	2	6.3%
	No	4	12.5%
5 to 10 years	Yes	6	18.8%
	No	5	15.6%
> 10 years	Yes	8	25.0%
	No	2	6.3%
Missing		5	15.6%
Total		32	100%

**Fig. 1:**
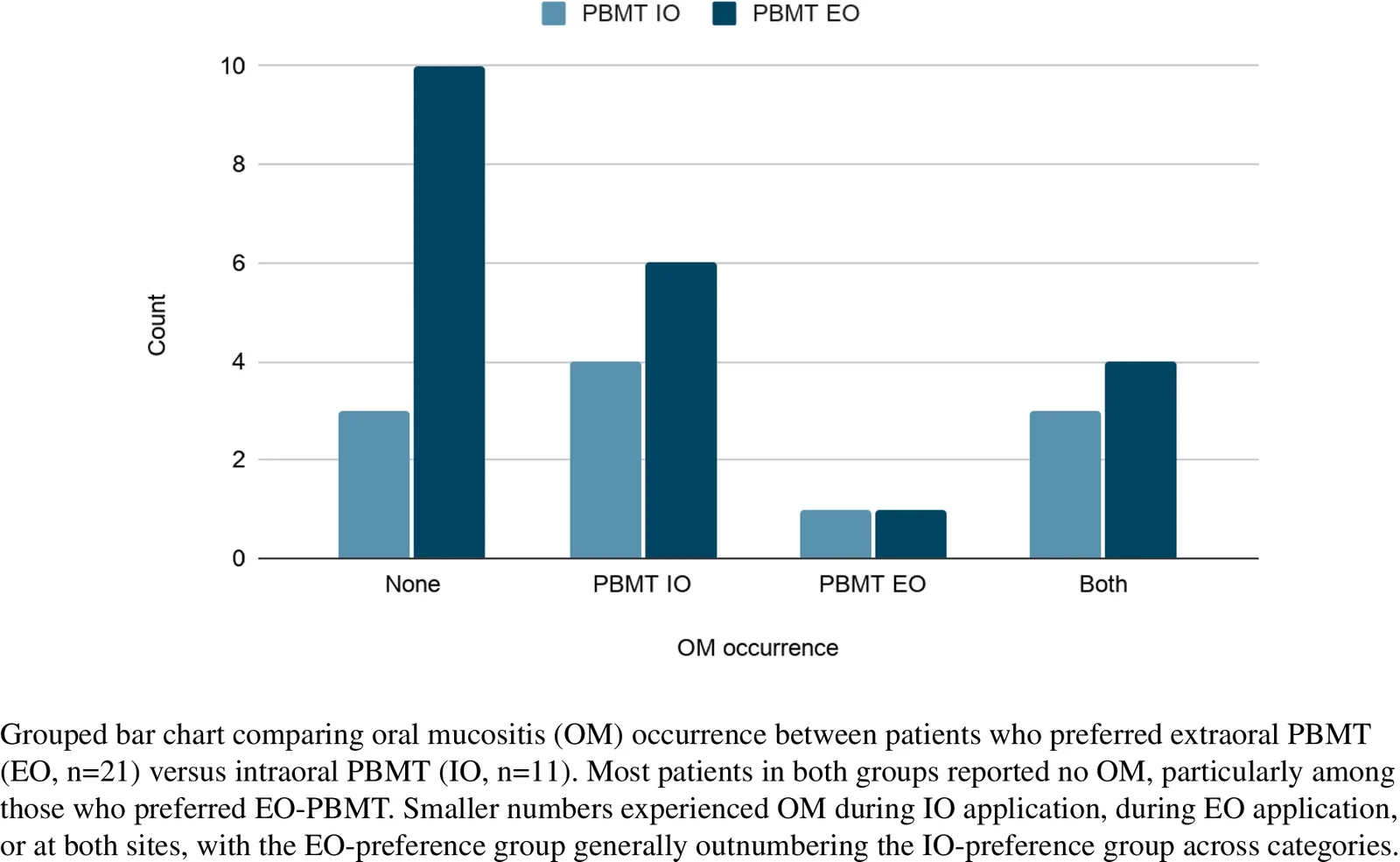
Patients’ preference of the PBMT modality with respect to oral mucositis

Regarding PBMT modality preference, 65.6% of respondents preferred EO-PBMT, whereas 34.4% preferred IO-PBMT. When asked which modality was associated with more severe OM, 28.1% reported worse outcomes with IO-PBMT, 18.8% with EO-PBMT, and 15.6% perceived no difference between modalities (Fig. 1).

**Fig. 2:**
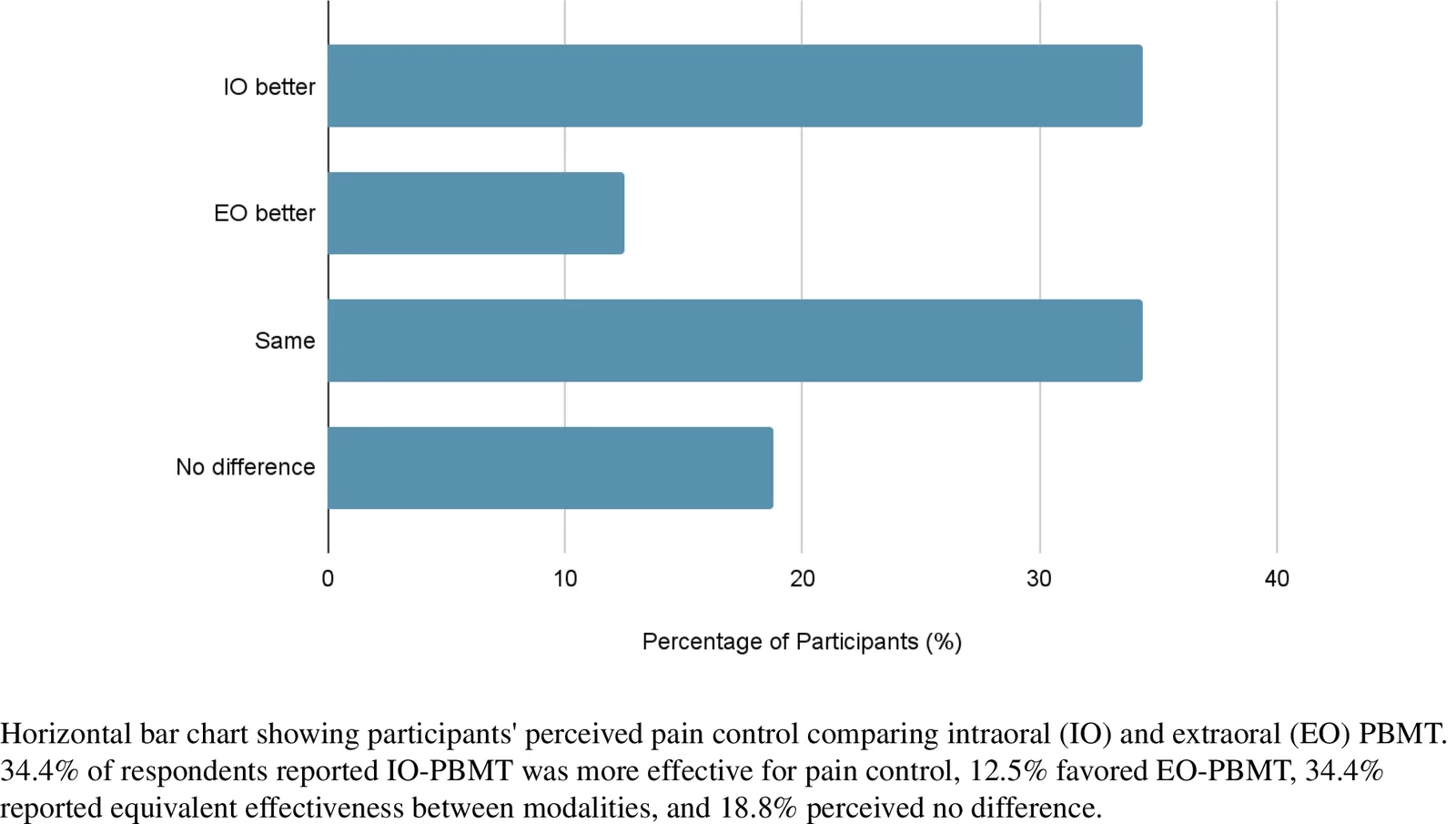
Perceived pain control

In terms of perceived pain control, 34.4% indicated that IO-PBMT was more effective, 12.5% favored EO-PBMT, 34.4% reported equivalent effectiveness, and 18.8% perceived no difference (Fig. 2) (*p* = 0.244).

**Fig. 3:**
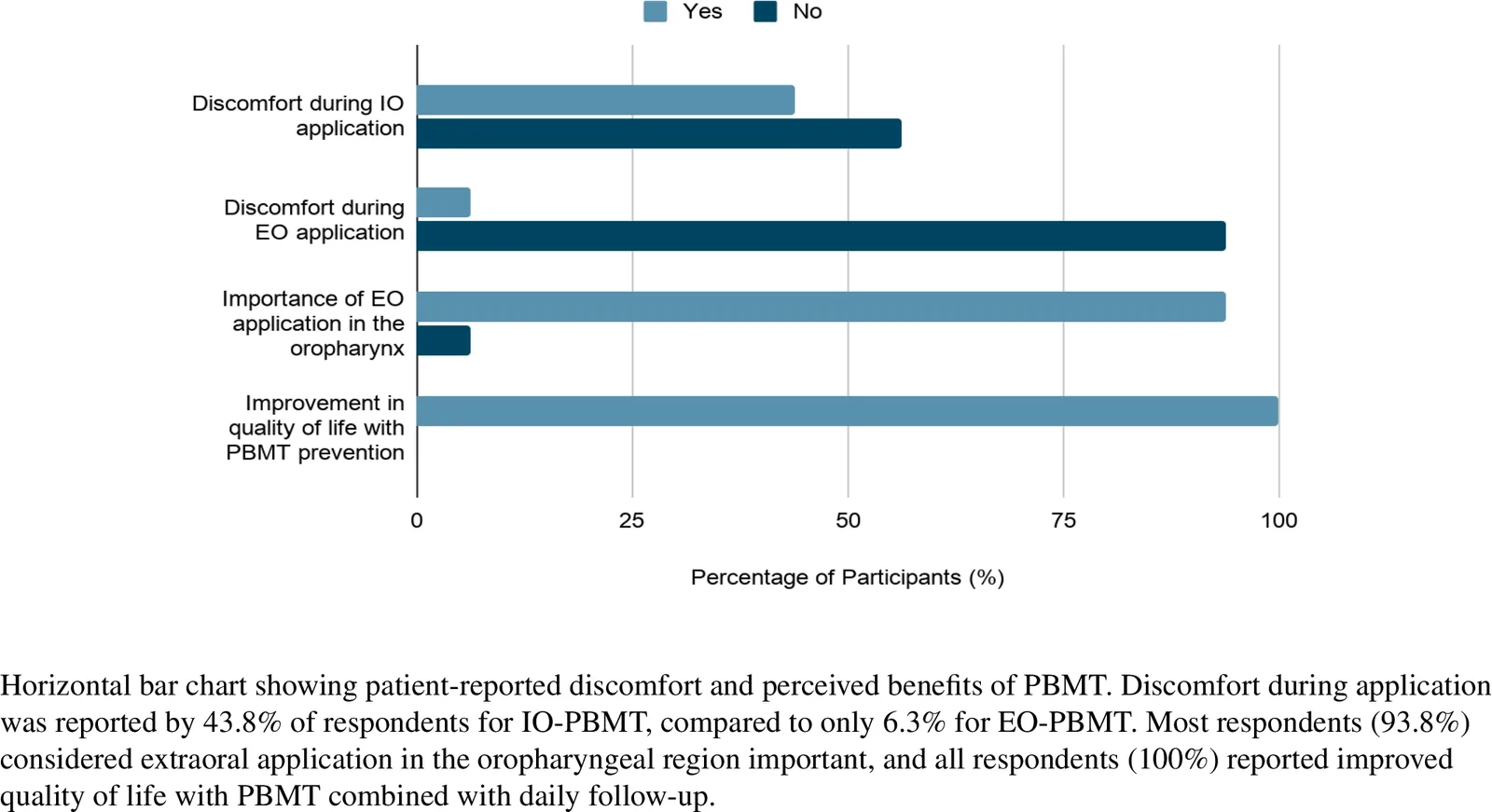
Patient-reported discomfort and perceived benefits of intraoral and extraoral PBMT

Discomfort during application was reported by 43.8% of respondents during IO-PBMT, compared to 6.3% during EO-PBMT, demonstrating a statistically significant difference in discomfort perception between the modalities (McNemar test, *p* = 0.001). Most respondents (93.8%) considered the possibility of extraoral application in the oropharyngeal region important. All respondents reported that PBMT combined with daily follow-up improved the patient’s quality of life (Fig. 3). Paired comparisons of reported discomfort between modalities are presented in Table 3.

**Table 3:** Paired comparison of reported discomfort during intraoral and extraoral PBMT

	EO-PBMT discomfort: yes		EO-PBMT discomfort: no		Total		*p* value McNemar test
	*N*	%	*N*	%	*N*	%	
IO-PBMT discomfort: yes	1	7.1%	13	92.9%	14	100%	
IO-PBMT discomfort: no	1	5.6%	17	94.4%	18	100%	**0.001**
Total	2	6.2%	30	93.8%	32	100%	

No statistically significant association was observed between PBMT preference (EO or IO PBMT) and the occurrence of OM, regardless of the definition used (Fisher’s exact test; *p* = 0.635; *p* = 0.694; *p* = 0.411). Similarly, no significant association was found between preferred modality and perceived effectiveness for pain control (*p* = 0.244).

A borderline association was observed between PBMT preference and discomfort during IO-PBMT (p = 0.061), with a higher proportion of discomfort reported among those who preferred EO-PBMT. No association was observed between EO-PBMT discomfort and modality preference (*p* = 1.000), indicating that factors other than discomfort may have influenced the preference for EO-PBMT.

A statistically significant association was found between PBMT modality preference and the reasons reported for this choice (*p* = 0.032). Respondents who preferred EO-PBMT primarily cited lower discomfort and the advantage of not requiring mouth opening.

## Discussion

Oral mucositis (OM) remains one of the most frequent and debilitating complications of antineoplastic therapy in pediatric patients, with significant impact on quality of life [22]. OM may lead to treatment interruptions, prolonged hospital stays, and increased healthcare costs [23, 24]. Children are particularly susceptible to this condition because of their higher epithelial mitotic rate [25]. In addition, pediatric oncology protocols often include intensive chemotherapy regimens involving agents associated with OM development, such as methotrexate [26]. As a result, the incidence of OM in this population ranges from 40 to 81% [22].

PBMT has emerged as a promising approach for OM prevention and management. Previous evidence indicates that PBMT can reduce OM severity and duration, while also alleviating associated symptoms such as pain and discomfort [22, 27]. In this study, a clear pattern emerged: extraoral PBMT was associated with higher acceptance and tolerability, whereas intraoral PBMT was more frequently perceived as more effective for pain control. Unlike the primary clinical outcomes of the randomized clinical study, the present study specifically explored caregiver-reported outcomes related to the acceptability and perceived effectiveness of the two PBMT delivery approaches.

The preference for PBMT-EO observed in 65.6% of participants appears to be mainly related to greater comfort during application and the fact that the procedure does not require opening the oral cavity. This interpretation is supported by the paired comparison between modalities, which showed a significantly lower frequency of discomfort during EO-PBMT than during IO-PBMT (*p* = 0.001). This is particularly relevant in pediatric patients, who may present with trismus, pain, or limited cooperation during OM episodes [28, 29]. Similar findings were reported in adult patients, with greater comfort associated with extraoral application [30].

Previous studies have demonstrated favorable outcomes with PBMT delivered on consecutive days as well as on alternate days, suggesting that different frequency regimens may be effective in OM management [31–34]. In the present study, PBMT was administered daily for 3 to 6 days, in accordance with protocols described in the literature [21, 35]. Although PBMT has been widely studied in different application regimens, the present findings highlight that the need for repeated PBMT applications over several days makes treatment tolerability and comfort central aspects, especially in pediatric patients [29]. Therefore, beyond clinical efficacy, acceptance of the method by patients and families should be considered a relevant factor in protocol selection and standardization.

A statistically significant association was identified between the preferred type of laser and the reasons for this choice (*p* = 0.032), as well as a clear trend toward greater discomfort with the intraoral technique. Rather than overstating this finding, it is more appropriate to interpret it as evidence that comfort-related aspects may strongly influence treatment preference in this setting.

The literature on PBMT for OM prevention and treatment remains heterogeneous and lacks standardization, particularly in pediatric populations [29, 33, 36]. Although current guidelines recommend intraoral PBMT, its application is limited by the need for patient cooperation and adequate visualization of the application field [27].

In addition, the intraoral route has important operational limitations. It is more time-consuming, requires application at multiple points across the oral mucosa, and may increase chair time and affect hospital logistics [18]. These factors may compromise comfort and adherence, particularly in pediatric patients, in whom maintaining mouth opening for prolonged periods can be challenging. Furthermore, because pediatric oral mucosa has distinct biological characteristics and children differ in their tolerance to time-consuming procedures, PBMT protocols must be appropriately adapted for this population [36]. In the absence of standardized protocols or evidence of superiority, Tomaževič et al. (2020) suggested that the most appropriate PBMT parameter may be the one that is fastest and most comfortable for the patient [33].

From this perspective, the clinical practicality of PBMT-EO deserves particular attention. The review by Adnan et al. (2021) described two studies evaluating the applicability of PBMT-EO in pediatric inpatient units [17]. In the first, involving 10 patients aged 4 to 21 years, successful daily prophylactic PBMT-EO was reported, with no pain and no need for treatment interruption [32]. In the second, a combined PBMT-IO and PBMT-EO protocol was used therapeutically in 22 patients aged 3 to 18 years with OM. Success was observed in 77% of episodes, and the procedures were well tolerated, with no reported adverse events [34]. More recently, no relevant differences were observed between PBMT-EO and PBMT-IO regarding mucositis severity, lesion duration, or functional impairment, suggesting similar efficacy between the two approaches [37].

Taken together, these studies support the safety and feasibility of the extraoral modality in pediatric patients [30, 37]. In the present study, PBMT-IO required irradiation of 42 points, resulting in a total treatment time of 20 to 40 min, whereas PBMT-EO was applied to only three anatomical regions, with simultaneous emission at 24 points, reducing treatment time to approximately 5 to 10 min. In addition to being faster, extraoral application avoids direct contact with mucositis lesions, which may reduce the risk of opportunistic infections and simplify clinical management, especially in pediatric patients who are less cooperative [17, 29]. Thus, PBMT-EO may represent a more feasible alternative in clinical settings where appointment time and procedural tolerability are limiting factors.

At the same time, some divergent findings between the two modalities warrant attention. A discrepancy was observed between procedural comfort and perceived analgesic efficacy: although PBMT-EO was preferred by most participants because of greater comfort, PBMT-IO was more frequently perceived as superior for pain control. One possible explanation lies in the dosimetry used, since the same parameters were applied to both groups. In extraoral application, light must traverse multiple tissue barriers, which may lead to greater energy dissipation and potentially reduce its analgesic effect [17]. Furthermore, differences in oral mucositis severity and distribution may have confounded pain perception and analgesic response.

On the other hand, PBMT-IO was also associated with greater discomfort during application, possibly because it requires mouth opening and manipulation of areas already sensitized by mucositis [17, 29]. In the present study, 50% of the sample developed OM during follow-up; and no statistically significant difference was observed between PBM modality and OM incidence. However, given the small sample size and the considerable proportion of missing OM data (15.6%), this finding should be interpreted as exploratory and hypothesis-generating, rather than as evidence of comparable clinical efficacy between the two modalities.

This finding is particularly relevant considering that most patients were between 5 and 10 years of age (34.4%). In this age group, assessment of quality of life and self-reported pain is challenging because of developmental, cognitive, and communication limitations. Although patients are generally the best informants of their own health-related quality of life, proxy reporting becomes especially important in pediatrics [38]. In this context, the perceptions of parents or caregivers represent a valuable complementary measure to guide supportive and palliative care strategies.

In adults, MASCC/ISOO guidelines strongly recommend intraoral PBMT to prevent OM in patients undergoing head and neck radiotherapy and hematopoietic stem cell transplantation [11]. However, in pediatric populations, there are still no consolidated protocols, particularly for extraoral application. The available evidence remains limited and heterogeneous [29, 33, 36], reinforcing the need for methodologically robust clinical trials to define safe and effective parameters for this population.

It is important to emphasize that the perceived efficacy evaluated in this study reflects caregivers’ subjective impressions after observing both PBMT modalities throughout treatment. Therefore, these findings should not be interpreted as evidence of superior clinical efficacy or greater effectiveness in preventing oral mucositis, which was beyond the scope of the present manuscript.

Some methodological aspects should be considered when interpreting these findings. The present study was not prospectively registered and it relied on a small sample size that limits statistical power and may have contributed to the absence of significant associations in some analyses, while also restricting generalizability. As this study was not designed as a comparative efficacy trial and lacked a formal sample-size calculation, findings related to preference and tolerability should be interpreted as exploratory.

The use of questionnaires completed by parents or caregivers introduces the possibility of information bias, especially for subjective outcomes such as pain, discomfort, and quality of life, which may not fully reflect the child’s experience. Recall bias should also be considered, since responses involved retrospective comparisons between different treatment cycles. Finally, the clinical heterogeneity of the sample, including differences in age, oncologic treatment, and occurrence of OM, may have influenced the reported perceptions.

The order of PBMT modalities was not formally balanced or statistically analyzed, precluding assessment of potential period or carryover effects. The exact interval between chemotherapy cycles was also not systematically recorded for each patient, although it typically followed the institutional protocol of 3 weeks. Because caregiver-reported outcomes were collected retrospectively after both cycles, responses may have been influenced by recency effects or by the severity of oral mucositis experienced in the most recent cycle, rather than reflecting an unbiased comparison of each modality in isolation.

Despite these limitations, the findings provide relevant evidence regarding the acceptability and applicability of intraoral and extraoral PBMT modalities in pediatric clinical practice. Preferences of patients and caregivers were strongly related to comfort and practicality, whereas perceived analgesic efficacy more often favored the intraoral route. These results highlight a clinically relevant trade-off between tolerability and perceived pain control, and reinforce the importance of considering not only clinical effectiveness, but also patient experience, when selecting PBMT protocols. Future studies with larger samples and prospective designs are needed to strengthen the evidence base and support protocol standardization in pediatric oncology.

## Conclusion

Intraoral and extraoral photobiomodulation represent viable options for oral mucositis prevention in pediatric oncology. Consideration of practical aspects such as tolerability and ease of application may support clinical decision-making.

## Supplementary Information

Below is the link to the electronic supplementary material.

ESM 1 (DOCX 7.79 KB)

## Data Availability

The datasets generated and/or analyzed during the current study are not publicly available due to ethical and privacy restrictions involving patient data. Data are stored at the hosting institution and are available from the corresponding author on reasonable request, subject to approval by the relevant ethics committee.

## References

[1] Valer JB, Curra M, Gabriel AF, Schmidt TR, Ferreira MBC, Roesler R, Evangelista JMC, Martins MAT, Gregianin L, Martins MD (2021) Oral mucositis in childhood cancer patients receiving high-dose methotrexate: prevalence, relationship with other toxicities and methotrexate elimination. Int J Paediatr Dent 31(2):238–246. 10.1111/ipd.12718 32815183

[2] Kamsvåg-Magnusson T, Thorsell-Cederberg J, Svanberg A, von Essen L, Arvidson J, Mellgren K, Toporski J, Ljungman G (2014) Parents and children’s perceptions of distress related to oral mucositis during haematopoietic stem cell transplantation. Acta Paediatr 103:630–636. 10.1111/apa.12627Sonis S. T. (2004). The pathobiology of mucositis. Nature reviews. Cancer, 4(4), 277–284. 10.1038/nrc1318 PMC4286779 24612395

[3] Sonis ST (2004) The pathobiology of mucositis. Nat Rev Cancer 4(4):277–284. 10.1038/nrc1318 15057287

[4] Cheng KK, Lee V, Li CH, Yuen HL, Epstein JB (2012) Oral mucositis in pediatric and adolescent patients undergoing chemotherapy: the impact of symptoms on quality of life. Support Care Cancer 20(10):2335–2342. 10.1007/s00520-011-1343-1 22167295

[5] Elting LS, Cooksley CD, Chambers MS, Garden AS (2007) Risk, outcomes, and costs of radiation-induced oral mucositis among patients with head-and-neck malignancies. Int J Radiat Oncol Biol Phys 68(4):1110–1120. 10.1016/j.ijrobp.2007.01.053 17398022

[6] Mazhari F, Shirazi AS, Shabzendehdar M (2019) Management of oral mucositis in pediatric patients receiving cancer therapy: a systematic review and meta-analysis. Pediatr Blood Cancer 66(3):e27403. 10.1002/pbc.27403 30421549

[7] Koźmiński P, Halik PK, Chesori R, Gniazdowska E (2020) Overview of dual-acting drug methotrexate in different neurological diseases, autoimmune pathologies and cancers. Int J Mol Sci 21(10):3483. 10.3390/ijms21103483 PMC7279024 32423175

[8] Al-Dasooqi N, Sonis ST, Bowen JM, Bateman E, Blijlevens N, Gibson RJ, Logan RM, Nair RG, Stringer AM, Yazbeck R, Elad S, Lalla RV, Mucositis Study Group of Multinational Association of Supportive Care in Cancer/International Society of Oral Oncology (MASCC/ISOO) (2013) Emerging evidence on the pathobiology of mucositis. Support Care Cancer 21(7):2075–2083. 10.1007/s00520-013-1810-y 23604521

[9] Lalla RV, Bowen J, Barasch A, Elting L, Epstein J, Keefe DM, McGuire DB, Migliorati C, Nicolatou-Galitis O, Peterson DE, Raber-Durlacher JE, Sonis ST, Elad S, The Mucositis Guidelines Leadership Group of the Multinational Association of Supportive Care in Cancer and International Society of Oral Oncology (MASCC/ISOO) (2014) MASCC/ISOO clinical practice guidelines for the management of mucositis secondary to cancer therapy. Cancer 120:1453–1461. 10.1002/cncr.28592 PMC4164022 24615748

[10] Elad S, Cheng KKF, Lalla RV, Yarom N, Hong C, Logan RM, Bowen J, Gibson R, Saunders DP, Zadik Y, Ariyawardana A, Correa ME, Ranna V, Bossi P, Mucositis Guidelines Leadership Group of the Multinational Association of Supportive Care in Cancer and International Society of Oral Oncology (MASCC/ISOO) (2020) MASCC/ISOO clinical practice guidelines for the management of mucositis secondary to cancer therapy. Cancer 126:4423–4431. https://doi.org/10.1002/cncr.3310

[11] Abdalla-Aslan R, Bonomo P, Keefe D, Blijlevens N, Cao K, Cheung YT, Fregnani ER, Miller R, Raber-Durlacher J, Epstein J, Van Sebille Y, Kauark-Fontes E, Kandwal A, McCurdy-Franks E, Finkelstein J, McCarvell V, Zadik Y, Ottaviani G, Amaral Mendes R, Speksnijder CM, MASCC Mucositis Study Group (2024) Guidance on mucositis assessment from the MASCC Mucositis Study Group and ISOO: an international Delphi study. EClinicalMedicine 73:102675. 10.1016/j.eclinm.2024.102675 PMC11200283 38933098

[12] Calarga CC, Cotomácio CC, Simões A (2024) Photobiomodulation for oral mucositis management in pediatric patients: a systematic review. Lasers Med Sci 39(1):272. 10.1007/s10103-024-04221-w 39523254

[13] Miranda-Silva W, Gomes-Silva W, Zadik Y, Yarom N, Al-Azri AR, Hong CHL, Ariyawardana A, Saunders DP, Correa ME, Arany PR, Bowen J, Cheng KKF, Tissing WJE, Bossi P, Elad S, Mucositis Study Group of the Multinational Association of Supportive Care in Cancer/International Society for Oral Oncology (MASCC/ISOO) (2021) MASCC/ISOO clinical practice guidelines for the management of mucositis: sub-analysis of current interventions for the management of oral mucositis in pediatric cancer patients. Support Care Cancer 29(7):3539–3562. 10.1007/s00520-020-05803-4 33156403

[14] Moraes JJC, Queiroga AS, De Biase RCCG et al (2009) The effect of low level laser therapy in different wavelengths in the treatment of oral mucositis—proposal for extra-oral implementation. Laser Phys 19:1912–1919. 10.1134/S1054660X09170150

[15] Hodgson BD, Margolis DM, Salzman DE, Eastwood D, Tarima S, Williams LD, Sande JE, Vaughan WP, Whelan HT (2012) Amelioration of oral mucositis pain by NASA near-infrared light-emitting diodes in bone marrow transplant patients. Support Care Cancer 20(7):1405–1415. 10.1007/s00520-011-1223-8 21725826

[16] Thieme S, Ribeiro JT, Dos Santos BG, de Almeida ZR, Severo MLB, Martins MAT, Matté C, Martins MD (2020) Comparison of photobiomodulation using either an intraoral or an extraoral laser on oral mucositis induced by chemotherapy in rats. Supportive care in cancer: official journal of the Multinational Association of Supportive Care in Cancer 28(2):867–876. 10.1007/s00520-019-04889-9 31165336

[17] Adnan A, Yaroslavsky AN, Carroll JD, Selting W, Juliano AF, London WB, Sonis ST, Duncan CN, Treister NS (2021) The path to an evidence-based treatment protocol for extraoral photobiomodulation therapy for the prevention of oral mucositis. Front Oral Health 2:689386. 10.3389/froh.2021.689386 PMC8757848 35048034

[18] Nugent M, Bryant V, Butcher C, Fisher H, Gill S, Goranova R, Hiu S, Lindley L, O’Hara J, Oluboyede Y, Patterson J, Rapley T, Robinson T, Rousseau N, Ryan V, Shanmugasundaram R, Sharp L, Smith Whelan R, Stocken DD, Ternent L, Walker J et al (2022) Photobiomodulation in the management of oral mucositis for adult head and neck cancer patients receiving irradiation: the LiTEFORM RCT. Health Technol Assess 26:1–172. 10.3310/UWNB3375 PMC9761526 36484364

[19] Villa A, Vollemans M, de Moraes A, Sonis S (2021) Concordance of the WHO, RTOG, and CTCAE v4.0 grading scales for the evaluation of oral mucositis associated with chemoradiation therapy for the treatment of oral and oropharyngeal cancers. Support Care Cancer 29(10):6061–6068. 10.1007/s00520-021-06177-x 33788003

[20] Birnie KA, Hundert AS, Lalloo C, Nguyen C, Stinson JN (2019) Recommendations for selection of self-report pain intensity measures in children and adolescents: a systematic review and quality assessment of measurement properties. Pain 160(1):5–18. 10.1097/j.pain.0000000000001377 30180088

[21] Ranna V, Cheng KKF, Castillo DA, Porcello L, Vaddi A, Lalla R, Bossi P, Elad S (2019) Development of the MASCC/ISOO clinical practice guidelines for mucositis: an overview of the methods. Support Care Cancer 27:3933–3948. 10.1007/s00520-019-04891-1 31286227

[22] Cruz LB, Ribeiro AS, Rech A, Rosa LG, Castro CG Jr, Brunetto AL (2007) Influence of low-energy laser in the prevention of oral mucositis in children with cancer receiving chemotherapy. Pediatr Blood Cancer 48:435–440. 10.1002/pbc.20943 16862549

[23] Sonis ST (2011) Oral mucositis. Anticancer Drugs 22:607–612. 10.1097/CAD.0b013e3283462086 21709615

[24] Marques MT, Arêde LT, Rodrigues JVS, Takeshita WM, Garcia VG, de Molon RS, Theodoro LH (2025) Photobiomodulation as a preventive strategy for oral mucositis in pediatric oncology: a systematic review and meta-analysis. J Dent 162:106074. 10.1016/j.jdent.2025.106074 40889543

[25] El Bousaadani A, Eljahd L, Abada R, Rouadi S, Roubal M, Mahtar M (2016) Prevention and treatment of mucositis in children with oral cancers: practical recommendations. Cancer Radiother 20:226–230. 10.1016/j.canrad.2015.11.006 27032624

[26] Ritwik P (2018) Dental care for patients with childhood cancers. Ochsner J 18:351–357. 10.31486/toj.18.0061 PMC6292458 30559620

[27] Patel P, Robinson PD, Baggott C, Gibson P, Ljungman G, Massey N et al (2021) Clinical practice guideline for the prevention of oral and oropharyngeal mucositis in pediatric cancer and hematopoietic stem cell transplant patients: 2021 update. Eur J Cancer 154:92–101. 10.1016/j.ejca.2021.05.013 34252760

[28] Gandhi K, Datta G, Ahuja S, Saxena T, G Datta A (2017) Prevalence of oral complications occurring in a population of pediatric cancer patients receiving chemotherapy. Int J Clin Pediatr Dent.10(2):166–171. 10.5005/iD-iournals-10005-1428 PMC5571386 28890617

[29] Gueiros LA, Gobbo M, Santos-Silva AR, Merigo E, Miranda-Silva W, Fregnani ER, Ottaviani G, Kauark-Fontes E, Bensadoun RJ, Arany P (2024) Underexplored areas of photobiomodulation in oral oncology: an expert analysis. Photobiomodul Photomed Laser Surg 42:609–619. 10.1089/photob.2023.0155 39422592

[30] Klein IP, Pinto MBR, Só BB, de Farias Gabriel A, Mendonça NF, da Cruz Santos LB, Farias KM, Mores AL, Martins MAT, Silva ACPRE, Brandão TB, Santos-Silva AR, Alves FA, Martins MD (2025) Intraoral vs. extraoral photobiomodulation therapy for oral mucositis in head and neck cancer patients: a multicenter, randomized, single-blind clinical trial. Support Care Cancer 33:842. 10.1007/s00520-025-09877-w 40928526

[31] Gobbo M, Ottaviani G, Perinetti G, Ciriello F, Beorchia A, Giacca M, Di Lenarda R, Rupel K, Tirelli G, Zacchigna S, Biasotto M (2014) Evaluation of nutritional status in head and neck radio-treated patients affected by oral mucositis: efficacy of class IV laser therapy. Support Care Cancer 22:1851–1856. 10.1007/s00520-014-2155-x 24554204

[32] Treister NS, London WB, Guo D, Malsch M, Verrill K, Brewer J et al (2016) Feasibility study evaluating extraoral photobiomodulation therapy for prevention of mucositis in pediatric hematopoietic cell transplantation. Photomed Laser Surg 34:178–184. 10.1089/pho.2015.4021 26982624

[33] Tomaževič T, Potočnik U, Cizerl D, Jazbec J (2020) Optimization of photobiomodulation protocol for chemotherapy-induced mucositis in pediatric patients. Photobiomodul Photomed Laser Surg 38:466–471. 10.1089/photob.2019.4794 32678713

[34] Noirrit-Esclassan E, Valera MC, Vignes E, Munzer C, Bonal S, Daries M et al (2019) Photobiomodulation with a combination of two wavelengths in the treatment of oral mucositis in children: the PEDIALASE feasibility study. Arch Pediatr 26:268–274. 10.1016/j.arcped.2019.05.012 31281038

[35] Nunes LFM, de Arruda JAA, Souza AF, Silva RCC, Lanza CRM, Kakehasi FM, Mesquita RA, Abreu LG, Travassos DV, Silva TA (2020) Prophylactic photobiomodulation therapy using 660 nm diode laser for oral mucositis in paediatric patients under chemotherapy: 5-year experience from a Brazilian referral service. Lasers Med Sci 35:1857–1866. 10.1007/s10103-020-03060-9 32535807

[36] Hafner D, Hrast P, Tomaževič T, Jazbec J, Kavčič M (2023) Photobiomodulation for chemotherapy-induced oral mucositis in pediatric patients. Biomolecules 13:418. 10.3390/biom13030418 PMC10046229 36979353

[37] Gabriel A, Soares J, Kovalski L, Curra M, Pinto M, Wagner V, Gregianin L, Martins M, Alves F, Martins M (2025) Efficacy of extraoral photobiomodulation therapy in the management of oral mucositis in pediatric oncology patients: a preliminary study. Oral Surg Oral Med Oral Pathol Oral Radiol. 10.1016/j.oooo.2024.10.104

[38] Bradlyn AS (2004) Health-related quality of life in pediatric oncology: current status and future challenges. J Pediatr Oncol Nurs 21:137–140. 10.1177/1043454204264376 15296041

[39] Taylor ZL, Miller TP, Board SG, et al. (2025) What is the expected clearance of methotrexate? A therapeutic drug monitoring reference guide for high-dose methotrexate use in pediatric malignancies. Pediatr Blood Cancer. 10.1002/pbc.31744Digital Object Identifier (DOI)40254820

[40] Al Manasour M, Absi A, Alhuraiji A, et al. (2025) Consensus on managing delayed methotrexate elimination in high-dose therapy: insights from the Middle East. Front Oncol 15. 10.3389/fonc.2025.1660937 PMC12631234 41278262

