# Supplementary material for: Acceptance and perceived efficacy of intraoral versus extraoral photobiomodulation for oral mucositis prevention in pediatric patients
Source: Support Care Cancer. 2026 Sep 23;34(10):1007. doi: 10.1007/s00520-026-11227-3 (PMC13601188; doi:10.1007/s00520-026-11227-3)
Supplement: Supplementary file 1 [file 520_2026_11227_MOESM1_ESM.docx]

**SUPPLEMENTARY MATERIAL – Questionnaire on Laser Therapy and Oral Mucositis**

Hello!
 We developed this questionnaire to learn more about the devices we use to prevent and treat oral mucositis.
 Through it, we will collect some information that we intend to publish in a scientific journal to help other hospitals and professionals improve laser therapy protocols using different devices.

Please note that the **intraoral laser** is the device used **inside the mouth**, while the **extraoral laser** is the larger device used **outside the mouth**.

These questions are addressed to the patient, but parents or guardians may assist in answering according to their perception.
 Your identity will remain confidential at all times.

**Email:**
 **Do you agree to answer this questionnaire?**
 ☐ Yes  ☐ No

**What is your name?** ___________________________________________________________________

**Which laser did you prefer?**
 ☐ Intraoral (inside the mouth)  ☐ Extraoral (outside the mouth)

**Why did you prefer the laser selected above?** *(You may choose more than one answer)*
 ☐ It is faster
 ☐ It causes less discomfort
 ☐ I don’t need to open my mouth
 ☐ I don’t feel nauseated during the application

**Did you develop oral mucositis during any of the applications?**
 ☐ Intraoral (inside the mouth)
 ☐ Extraoral (outside the mouth)
 ☐ Both
 ☐ None

**In your opinion, with which device was the mucositis worse?**
 ☐ Intraoral (inside the mouth)
 ☐ Extraoral (outside the mouth)
 ☐ The same
 ☐ I did not have mucositis

**Did you feel any discomfort during the intraoral laser application?**
 ☐ Yes  ☐ No

**Did you feel any discomfort during the extraoral laser application?**
 ☐ Yes  ☐ No

**Which laser device was more effective in controlling your pain?**
 ☐ Extraoral  ☐ Intraoral  ☐ Both equally  ☐ No difference noticed

**In your opinion, did the laser application and daily follow-up improve your quality of life?**
 ☐ Yes  ☐ No  ☐ No difference noticed

**Do you think that using the extraoral laser makes it possible to apply the light to the throat (oropharynx)?**
 ☐ Yes  ☐ No
